# Ultrasound-responsive highly biocompatible nanodroplets loaded with doxorubicin for tumor imaging and treatment *in vivo*

**DOI:** 10.1080/10717544.2020.1739170

**Published:** 2020-03-13

**Authors:** Xiaoying Zhou, Lu Guo, Dandan Shi, Dong Meng, Xiao Sun, Mengmeng Shang, Xinxin Liu, Yading Zhao, Jie Li

**Affiliations:** a Department of Ultrasound, Qilu Hospital of Shandong University, Jinan, China;; b The Key Laboratory of Cardiovascular Remodeling and Function Research, Chinese Ministry of Education, Chinese Ministry of Health and Chinese Academy of Medical Sciences, Qilu Hospital of Shandong University, Jinan, China

**Keywords:** High biocompatibility, drug delivery system, chitosan nanodroplets, contrast-enhanced ultrasound imaging, spatiotemporally controlled delivery

## Abstract

As an injectable anticancer drug delivery system, the biological safety of nanocarriers is the most important prerequisite for their clinical application. The objective of our study was to synthesize special ultrasound-responsive highly biocompatible chitosan nanodroplets (BCNDs), observe their spatiotemporally control the delivery of doxorubicin (DOX) *in vivo*. The experimental results showed that the BCNDs were successfully prepared with high biosafety *in vivo* and great ultrasound imaging ability. DOX-BCNDs promoted the anticancer effects of DOX *in vivo* and inhibited the development of tumors. They also reduced the side effects to the heart and kidneys. In conclusion, BCNDs are a new type of smart nanocarrier with high biocompatibility and efficacy have great potential to be used in the clinic.

## 1. Introduction

A growing number of studies on drug-loaded nanosystems have demonstrated the potential importance of their role in cancer treatment. The advantage of the nanodrug delivery system lies in its ability to deliver concentrated doses of drugs to specific areas, targeting tumor tissues *in vivo*. The nanodrug delivery system not only increases the local effect of drugs on tumors but also reduces the side effects of chemotherapeutic drugs entering other tissues in the process of blood circulation. However, most nanodrug delivery systems still face some challenges in clinical practical applications. The first challenge is how to increase intracellular drug release when the nanodrug delivery system aggregates and adheres to tumor cells. Previous experiments have shown that most relatively large nanoparticles enter cells by endocytosis (Zhang et al., 2015; Spangler et al., 2016). However, some traditional delivery vehicles such as symmetrical polymer vesicles are slow or inefficient (Liu et al., 2014). And cell uptake efficiency is usually influenced by concentration, temperature, and other factors (Wang et al., 2014). Another route of entry is through receptor-mediated ingestion. However, the significant heterogeneity within or between tumor types has been revealed as a formidable barrier for nanodroplets to act effectively (Yin et al., 2014). Second, the treatment process is not spatiotemporally controlled. Therefore, it is impossible to further evaluate and improve the treatment plan over the course of treatment. Some self-powered drug delivering systems and the sensors for microfluidics strategy designed to solve this problem (Nie et al., 2018; Liu et al., 2019). Ultrasound-guided drug delivery systems can result in reversible permeabilization of the plasma membrane of cells through ultrasound-targeted microbubble destruction (UTMD), enabling drugs to effectively enter tumor cells. UTMD, with the advantages of noninvasiveness, low immunogenicity and toxicity, repeatability and temporal and spatial target specificity, is an accurate and visual treatment with simultaneous ultrasound images (Liao et al., 2014).

Based on their importance, ultrasound-guided drug delivery systems are becoming a hotspot in targeted therapy research (Tang et al., 2018). To penetrate the endothelial cell gap of neovascularization more easily, the particle size of ultrasound contrast agents has gradually decreased from the range of microns to nanometers. With increasing research and clinical applications of nanoultrasound contrast agents, their safety has attracted much attention (Krefting, 2009). Biosafety must be established before these agents can be used in clinical practice. Among all materials for nanoultrasound contrast agents, protein materials may cause allergic reactions (Chicken et al., 2007). Many stationary agents or surfactants, such as glutaraldehyde and tweens, are harmful to humans, while macromolecular materials pose potential risks to human safety (Zeiger et al., 2005; Li et al., 2015). Therefore, there is an urgent need to prepare nanoscale drug-loaded ultrasound contrast agents with high safety and definite efficacy.

Ultrasonic response nanobubbles synthesized by chitosan, perfluorohexane and other natural materials in our previous research has been proved to have higher biosafety than other contrast agents in cell experiments (Meng et al., 2019; Zhou et al., 2019). They may have a broad prospect in clinical application. Other study showed that replacing inert gas with perfluorohexane can make the structure of the ultrasound contrast agents more stable (Abou-Saleh et al., 2016). In this study, BCNDs were synthesized using perfluorohexane instead of perfluoropropane. Other materials remained unchanged. A high-dose acute toxicity test was used to verify the safety of BCNDs *in vivo*. At the same time, these highly biocompatible nanodroplets were used for drug delivery *in vivo* for the first time. Doxorubicin (DOX) a well-known anticancer drug with significant cardiotoxicity and nephrotoxicity, was chosen as a model drug for loading. We observed the imaging effects *in vivo* and analyzed the value of DOX-BCNDs in improving the therapeutic index of DOX and overcoming its side effects. Furthermore, we revealed the causes of these phenomena using *in vivo* small animal imaging systems.

## 2. Materials and methods

### 2.1. Chemicals

DOX was obtained from Sigma Aldrich (St. Louis, MO, USA). Epikuron 200 (soy lecithin containing 95% dipalmitoyl phosphatidylcholine) was provided by Lukas Meyer, (Hamburg, Germany). Chitosan was sourced from Bozhihuili (Qingdao, China). Perfluorohexane (PFH) was supplied by Macklin Biochemical (Shanghai, China). Pluronic F68 was purchased from Sigma Aldrich and was also used in this study. All other chemicals were of analytical grade.

### 2.2. Animals

All animal care and experimental protocols complied with the Animal Management Rules of Ministry of Health of People’s Republic of China (document No 55, 2001). Six- to eight-week-old male BALB/c mice were adopted by Pengyue Laboratory Animal Breeding Company (Shandong, China). The animals were kept in cages with free access to food and water under 12 h light-dark cycles. To establish the development of solid tumors, diluted ascites containing H22 cells (100 μl/mouse) were injected subcutaneously into the left forelimb armpit with a very fine needle. Viable cells were counted and adjusted to a concentration so that tumors appeared at the injection site one week after transplantation.

### 2.3. Synthesis of BCNDs

The BCND shell was composed of chitosan, lecithin and palmitic acid. The core of the BCNDs was liquid perfluorohexane. The appropriate dose of chitosan was dissolved in ultrapure water to prepare a solution for use. Epikuron 200 (0.02 g) was dissolved in a certain proportion of ethanol solution. Another 0.005 g of palmitic acid was dissolved in ultrapure water and bathed at 70 °C until completely dissolved. The palmitic acid solution was mixed with Epikuron 200 solution and homogenized with ultrapure water of the appropriate volume. Subsequently, the palmitic acid-Epikuron 200 solution was added to the prepared chitosan solution and homogenized again. An appropriate amount of perfluorohexane solution was added to the mixed solution, which was mixed evenly. All of the mixed solutions were shaken with an ultrasound cell breaker for 1 min (30% output power). The final step was the addition of an appropriate amount of Pluronic F68 into the BCND suspension.

### 2.4. Characterization and stability of BCNDs

The suspension of BCNDs was diluted by adding an appropriate amount of deionized water. The average particle size (hydrodynamic diameter, nm) and ζ-potential of the BCNDs were measured by a Delsa Nano C particle size and ζ-potential analyzer (Beckmann, Fullerton, CA, USA). All measurements were performed in triplicate to calculate the mean value. The shape of the BCNDs was then observed and imaged under an optical microscope (Olympus, Tokyo, Japan)). To evaluate the stability of the BCNDs, they were stored in a refrigerator at 4 °C for 24 h or incubated in human serum (Seronorm™ Human, Norway) at 37 °C for 1 h. The morphology and size of the BCNDs were also observed by optical microscopy.

### 2.5. *In vivo* biosafety testing

Referring to previous literature (Zhang et al., 2008), to test the biological safety, high-dose BCNDs (80 mg/kg total dose and 0.5 ml of administration volume) was injected into the tail vein of the mice. The control group mice were injected with the same dose of saline intravenously. All experimental animals were fasted for 12 h before the experiment. The general situations of the mice in each group were observed. The weights of mice were recorded on the 0 day, 7th day, and 14th day. Blood biochemical tests were performed on the 14th day after treatment, and HE was performed on the dissected heart, liver, spleen, lung, and kidney.

### 2.6. Determination of entrapment efficiency (EE) and loading efficiency (LE) of doxorubicin-loading DOX-BCNDs

To prepare DOX-BCNDs, DOX was added to the prepared nanodroplet suspension, shaking slowly for 20 min. Then, DOX-BCNDs were obtained by centrifugation. The amount of entrapped DOX in DOX-BCNDs was determined by centrifuging the nanodroplet solution and measuring the absorbance of DOX in the supernatant with a UV–vis spectrophotometer at 480 nm (UV-2450, Shimadzu, Japan). DOX-BCNDs were imaged under a fluorescence microscope (Nikon TE2000-S, Tokyo, Japan) equipped with a 100 × oil-immersion objective lens.

### 2.7. Ultrasound imaging of DOX-BCNDs *in vitro* and *in vivo*

The *in vitro* ultrasound imaging device is shown in Figure 4 (Duan et al., 2017). DOX-BCNDs were diluted with PBS and placed in the device. After smearing the contrast agent on the M9L probe of the GE ultrasound instrument, the probe was placed on the side of the development bag and imaged under specific ultrasound parameters. In the *in vivo* imaging experiments, we selected a tumor-bearing mouse and performed local hair removal at the prominent part of the tumor and surrounding areas, injected 0.1 ml of DOX-BCND suspension into the tumor, and then performed ultrasound imaging immediately. The experiment also used a GE ultrasound unit to develop small organ probes. The parameters of the ultrasound were set at a depth of 3 cm and a mechanical index (MI) of 0.22.

### 2.8. *In vivo* fluorescence imaging

Twenty tumor-bearing BALB/c mice were randomly divided into DOX-BCND groups and DOX groups (10 mice in each group). The hair on the tumor tissue and peripheral tissue was removed from all mice. DOX-BCNDs (0.1 ml) or the same dose of DOX was injected into each tumor. The tumor areas were immediately subjected to ultrasonic irradiation for 1 min after injection (output power density of 2 W/cm^2^) in the DOX-BCNDs group. All mice were then anesthetized with inhaled isoflurane and placed into IVIS kinetic small animal imaging systems. All procedures were carried out under light hindered conditions. Exciting fluorescence was performed and photographs were taken at 0 h, 2 h, 4 h, and 12 h after injection. The local concentration of DOX in the tumor was determined and compared between the groups. Twelve hours later, all the mice were sacrificed and the heart, liver, spleen, lung, kidney, and tumor tissues were dissected and compared by fluorescence imaging.

### 2.9. Tumor suppression experiments *in vivo*

Thirty-five BALB/c mice were randomly divided into five groups, including a control group, DOX group, DOX ultrasound group, DOX-BCNDs ultrasound group and double DOX-BCNDs ultrasound group. On the 7th day after implantation of the H22 tumor cells, local therapy was started in the mice. The mice in the DOX-BCNDs ultrasound group underwent three systemic injections of DOX-BCNDs (6 mg/kg) given on days 7, 9, and 11. The mice in the DOX group and the DOX ultrasound group were injected with the equivalent amount of DOX. PBS (0.1 ml) was injected into each tumor in the control group. In the DOX ultrasound group, DOX-BCND ultrasound group and double DOX-BCND ultrasound group, the tumor areas were immediately exposed to ultrasonic irradiation for 1 min after injection (output power density of 2 W/cm^2^).

The general situation of the mice in each group was observed. The mice were weighed at the beginning of the experiment and at the end of treatment. The body mass of experimental animals was weighed by electronic balance. The weight changes of the mice in each group were calculated. The tumor volumes of the mice bearing tumors were measured before and after treatment, the difference in the volume reduction was calculated, and the inhibition rate was calculated by the following formula.

V=L*W*T/2

where L, W, and T are the length, width, and thickness of the tumor, respectively. The normalized tumor size (*D_n_*) was calculated using the following equation:

$$D_{n}=\sqrt[{3}]{\frac{V}{V_{o}}}$$

where *V* and *V_o_* are the current and initial tumor volumes (*V_o_* is the tumor volume at the start of treatment).

The tumor growth inhibition rate (IR) was calculated according to Equation (Baghbani & Moztarzadeh, 2017):

$$IR(\%)=1-\frac{\left(V_{t}\right)}{V_{c}}\times 100$$

where *V_c_* is the tumor volume in the control group and *V_t_* is the tumor volume in the treated group.

### 2.10. Anatomy and blood collection of mice

On the 13th day of the experiment, eyeball blood from mice in each group was collected for blood biochemistry and routine blood examination to evaluate adverse reactions. Hematological analysis was carried out using an automatic cell counter (ABX-MICROS-60 cell counter Horiba, Inc.). The samples were evaluated for the following hematological parameters: number of white blood cells, ratio of lymph, monocytes and granulocytes, red blood cell count, and hemoglobin. Part of the collected blood was centrifugally separated into blood plasma. The blood levels of creatinine (CREA), blood urea nitrogen (BUN), alanine aminotransferase (ALT), aspartate aminotransferase (AST), total protein (TP), albumin (ALB), lactate dehydrogenase (LDH), and creatine phosphokinase (CK) were measured. All mice were sacrificed, and tumor tissues were dissected.

### 2.11. He staining, apoptotic test, and immunohistochemistry

Histopathological analysis was performed on the dissected tumors. Paraffin-embedded tumors were sectioned into slices of 5 μm thickness, mounted on a glass microscope slide and stained with hematoxylin and eosin (HE) for microscopic observations (Ti50; Nikon Corporation). Immunohistochemical (Ki67) and TUNEL analyses were performed according to the instructions of the kit. Apoptotic pictures were observed by fluorescence microscopy. Ki67 pictures were read by Image-Pro software and compared among groups.

### 2.12. Statistical analysis

All data collection was repeated at least three times, and the data are expressed as the mean ± standard deviation. The data were statistically analyzed with SPSS software (version 18.0; SPSS, Inc., IL, USA). One-way ANOVA followed by the Newman-Keuls test was used to evaluate the differences among the treatments. *p*-Values < .05 were considered statistically significant.

## 3. Results

### 3.1. Characterization of the BCNDs

**Figure 1.**
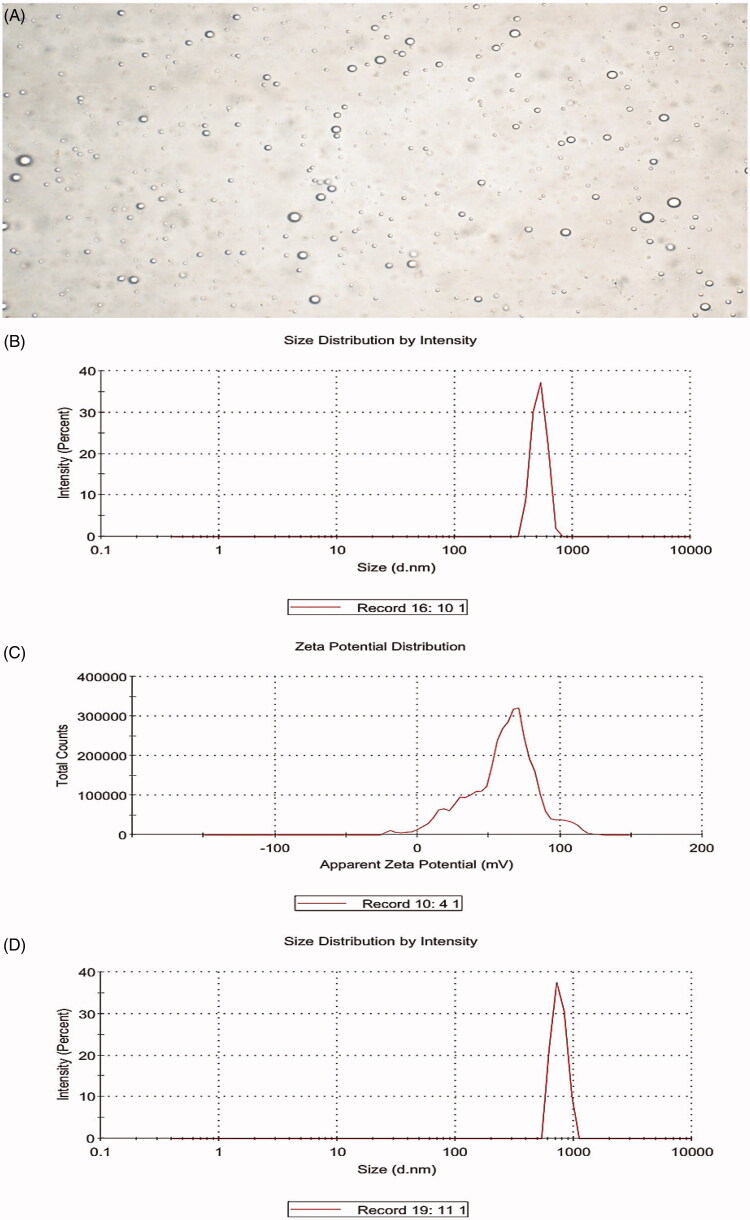
Characteristics of BCNDs. (A) Optical microscope images of BCNDs. (B) The size distribution of BCNDs. (C) The ζ potential of BCNDs. (D) The size distribution of BCNDs after incubation in human serum at 37 °C for 1 h.

The average diameter of the BCNDs was 519.6 ± 72.66 nm, the ζ-potential was 59.1 ± 24.1 mV, and the image of BCNDs was homogeneous under a microscope (Figure 1(A,C,D)). Microscopic images showed that the size of BCNDs remained unchanged after being placed at 4 °C for 24 or 48 h. After the BCND suspension was placed in human serum at 37 °C for 1 h, the average particle size of the BCNDs was slightly increased from 519.6 to 751.2 nm (Figure 1(D)).

### 3.2. High biosafety *in vivo*

**Figure 2.**
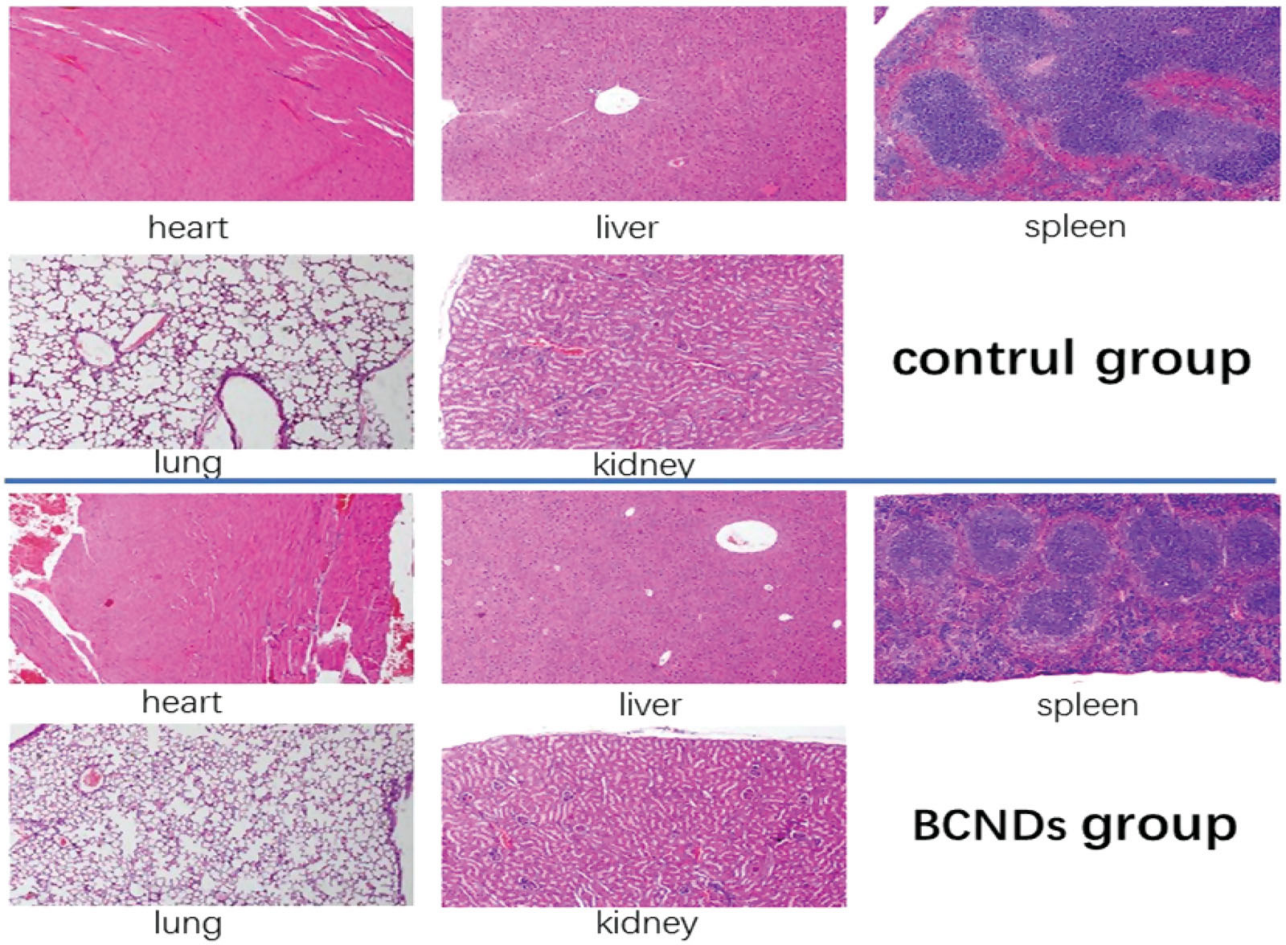
HE staining of paraffin sections in the two groups.

Twenty mice were randomly divided into two groups with 10 mice in each group. BCNDs were intravenously injected via the tail vein at a total dose of 80 mg/kg in the BCND group. Equivalent saline was used as a control. In the intravenous injection group of BCNDs, three mice showed temporary instability in standing on the day of administration and one mouse showed lethargy, all of which returned to normal the next day. There was no significant difference in body weights between the two groups (Table 1). No significant differences were found in any tested serum biochemical parameters between the two groups 14 days after intravenous injection (Table 2). Notably, BCND does not impair liver or kidney function. H&E staining of the heart, liver, spleen, and kidneys did not show any apparent change in cellular structures 14 days after BCND injection. In the BCND group, slight intra-alveolar hemorrhage was occasionally evident in lung tissues (Figure 2).

**Table 1.** Body weight of rats in the acute toxicity study of BCNDs (mean ± SD).

Group	Initial weight (g)	Weight 7 days after administration (g)	Weight 14 days after administration (g)
Control group	20.79 ± 1.18	27.78 ± 1.04	33.34 ± 1.27
BCNDs group	20.77 ± 1.02	27.30 ± 1.75	33.65 ± 1.82

**Table 2.** Biochemical test results (mean ± SD, *n* = 10).

Group	GLU (mmol/L)	BUN (mmol/L)	TBIL (mg/dL)	ALT (U/L)	AST (U/L)	TP (g/L)	ALB (g/L)	TG (mmol/L)	CHOL (mmol/L)
Control group	5.73 ± 0.28	8.14 ± 0.44	1.62 ± 0.15	39.52 ± 5.29	146.30 ± 16.93	65.57 ± 1.23	28.56 ± 0.50	1.32 ± 0.27	1.63 ± 0.22
BCNDs group	5.88 ± 0.68	8.27 ± 0.82	1.52 ± 0.16	42.56 ± 11.15	148.60 ± 20.25	65.15 ± 2.75	28.40 ± 1.58	1.36 ± 0.31	1.76 ± 0.20

There were no significant differences between the two groups (*p* > .05).

### 3.3. Ee and LE of DOX-BCNDs

**Figure 3.**
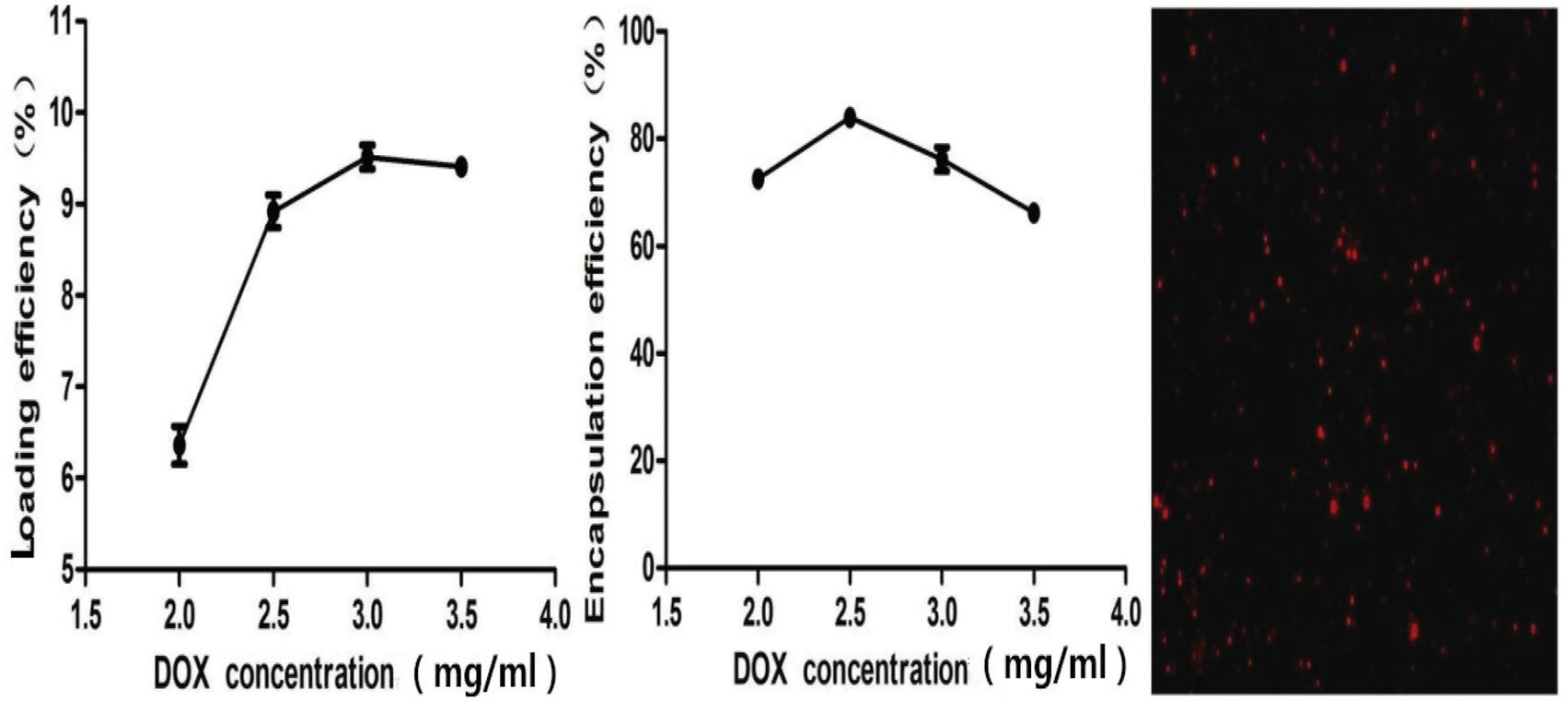
Influence of the DOX concentration on encapsulation efficiency and loading efficiency and fluorescence microscope images of BCNDs.

To determine the optimum DOX concentration in DOX-BCNDs, we added different initial doses of DOX to the BCND suspension and calculated the final concentration of DOX loaded on BCNDs by spectrophotometry at 480 nm. The effects of DOX concentration on the encapsulation efficiency (EE) and loading rate (LE) of DOX-BCNDs were studied. It can be seen from Figure 3 that when the DOX concentration is 2.5 mg/ml, the EE reaches its peak and then decreases slowly with a further increase in DOX dose, which may be due to near saturation of drug concentration. The LE reached its peak at 3 mg/ml and then declined. To select a relatively higher drug loading, we chose the 3 mg/ml concentration of DOX added in our experiment; the EE was 76.09%, and the LE was 9.51%. The DOX-BCNDs image under a fluorescence microscope is also shown in Figure 3.

### 3.4. Ultrasound imaging of DOX-BCNDs *in vitro* and *in vivo*

**Figure 4.**
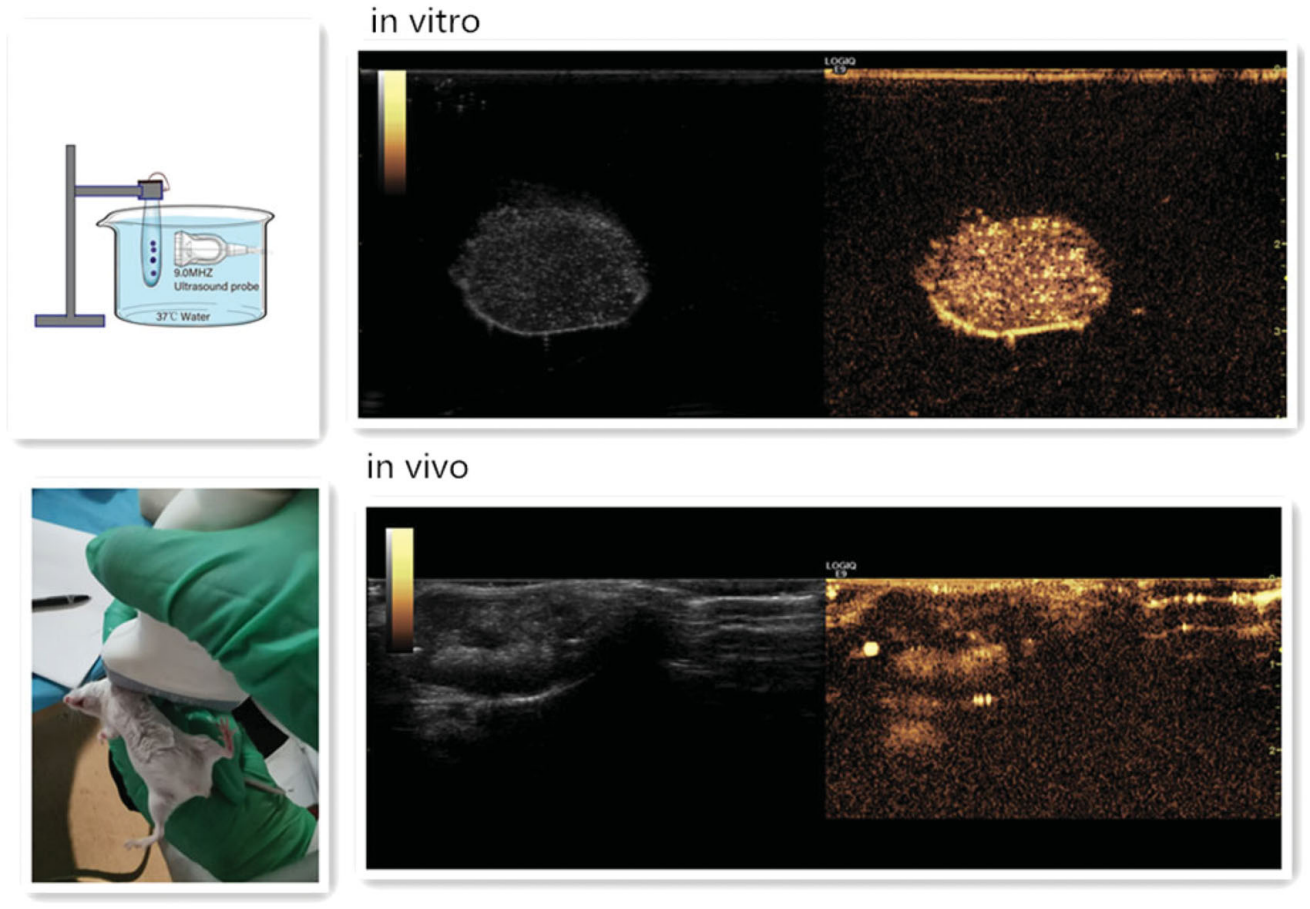
Ultrasound enhancement images of DOX-BCNDs *in vitro* and *in vivo*.

To evaluate the *in vitro* ultrasound imaging ability of DOX-BCNDs, experiments with DOX-BCNDs were carried out in a water bath at 37 °C. For *in vivo* imaging experiments, the DOX-BCNDs were injected directly into the implanted tumors of mice after 5 min in a 37 °C water bath. A clinical ultrasound scanner system was used. DOX-BCNDs showed satisfactory ultrasound enhancement both *in vivo* and *in vitro* (Figure 4).

### 3.5. *In vivo* fluorescence imaging of mice

**Figure 5.**
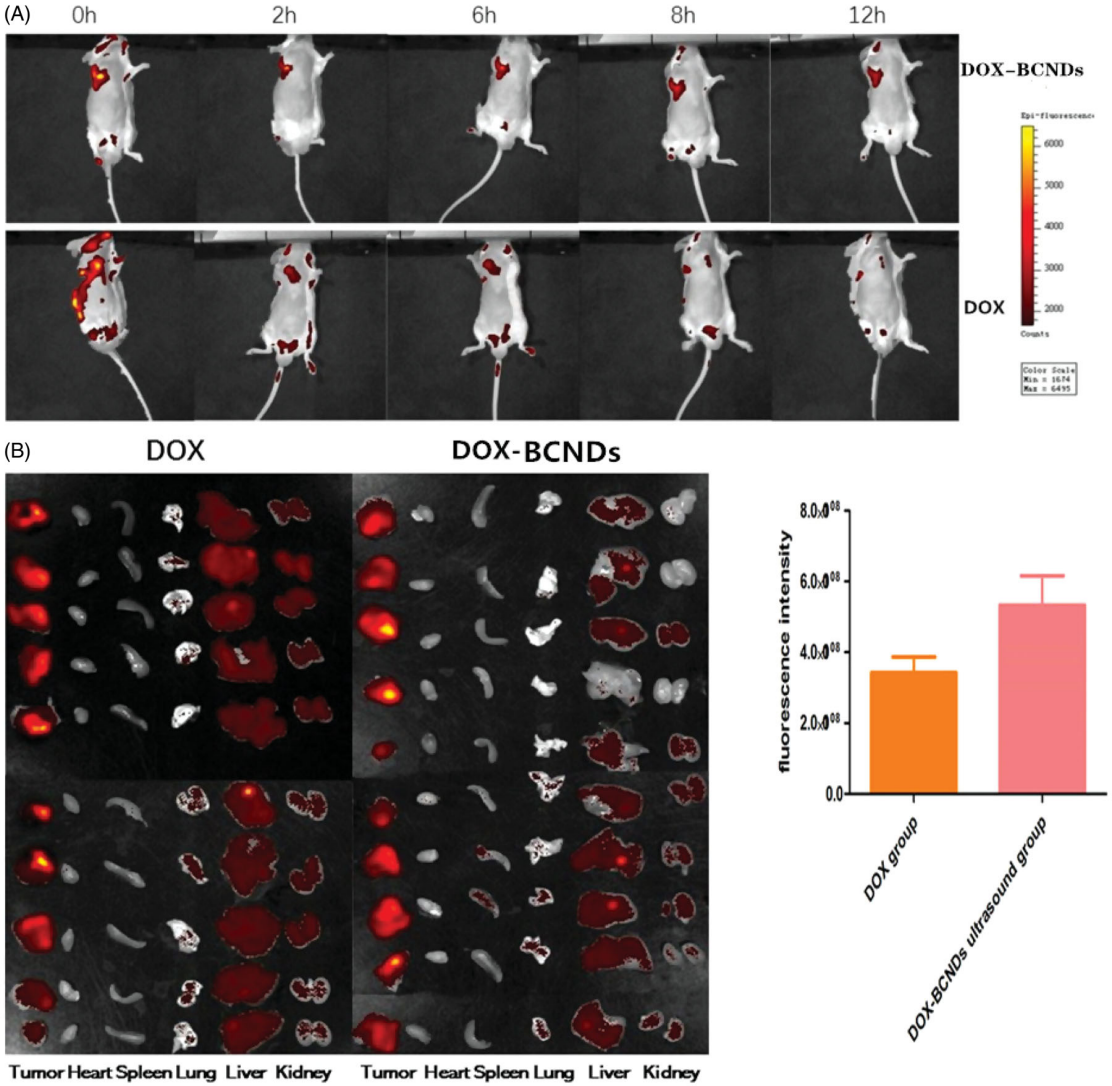
(A) *In vivo* imaging of DOX-BCNDs and DOX at different time points. (B) *In vivo* distribution of DOX in mice and comparison of fluorescence intensity in tumors between the two groups.

The local fluorescence attenuation of mice was observed at 0, 2, 6, 8, and 12 h after injection of DOX-BCNDS or the same dose of DOX. The DOX fluorescence in the tumors remained clear 12 h after injection in the DOX-BCNDs group (Figure 5(A)). Compared to the same dose in the DOX group, the local fluorescence intensity of the tumors decreased rapidly, and the fluorescence imaging was weaker after 12 h. Twelve hours later, all the mice in the two groups were sacrificed. Tumor, liver, heart, lung, spleen, and kidney tissues were removed for fluorescence imaging and analyzed by imaging system software. The results showed that the average fluorescence intensity of the tumors in the DOX-BCNDs group was higher than that in the DOX group. In Figure 5(B), we can also see that the amount of DOX entering the liver and kidneys through blood circulation was greater in the DOX group 12 h after injection.

### 3.6. Comparison of body weights and tumor volumes of mice after *in vivo* tumor treatment

To detect the therapeutic effect of DOX-BCNDs, 35 mice were randomly divided into five groups (*n* = 7). All of the mice in each group were tumorigenic on the seventh day after H22 cell implantation. During treatment, the mice in the DOX group, DOX ultrasound group, and DOX-BCNDs group were in good condition. Some mice in the control group and the double DOX-BCNDs group had poor food intake, decreased activity, or mental depression. No mice died in any of the groups.

The changes in body weight of the mice in each group were quite different (Table 3). The comparisons showed that the mice in the DOX group, DOX ultrasound group and DOX-BCNDs ultrasound group all gained weight. Among them, the weight gain of mice in the DOX-BCND ultrasound group was the most significant. The weights of the mice in both the control group and the double DOX-BCND ultrasound group decreased. These results suggest that DOX therapy may improve the survival status of mice with tumors to some extent. However, administration of DOX-BCNDs at an appropriate dose is also important.

**Table 3.** Comparison of the average changes in body weight and tumor volume of the mice in each group (mean ± SD).

Group	Weight change (g)	Tumor volume change (cm^3^)
Control group	–1.30 ± 0.82	0.74 ± 0.35
DOX group	0.60 ± 0.43**◆**	0.40 ± 0.17**◆**
DOX ultrasound group	0.12 ± 1.44**◆**	0.19 ± 0.36**◆**
DOX-BCNDs ultrasound group	1.30 ± 0.80**◆**	–0.24 ± 0.17◆*▼
Double DOX-BCNDs ultrasound group	–0.10 ± 1.89**▲**	–0.07 ± 0.14◆*
F	4.514	16.214
*p* Value	.006	<.001

Notes: **◆**Compared with the control group, *p* < .05; *Compared with the DOX group, *p* < .05; **▼**Compared with the DOX ultrasound group, *p* < .05; **▲**Compared with the DOX-BCNDs ultrasound group, *p* < .05.

**Figure 6.**
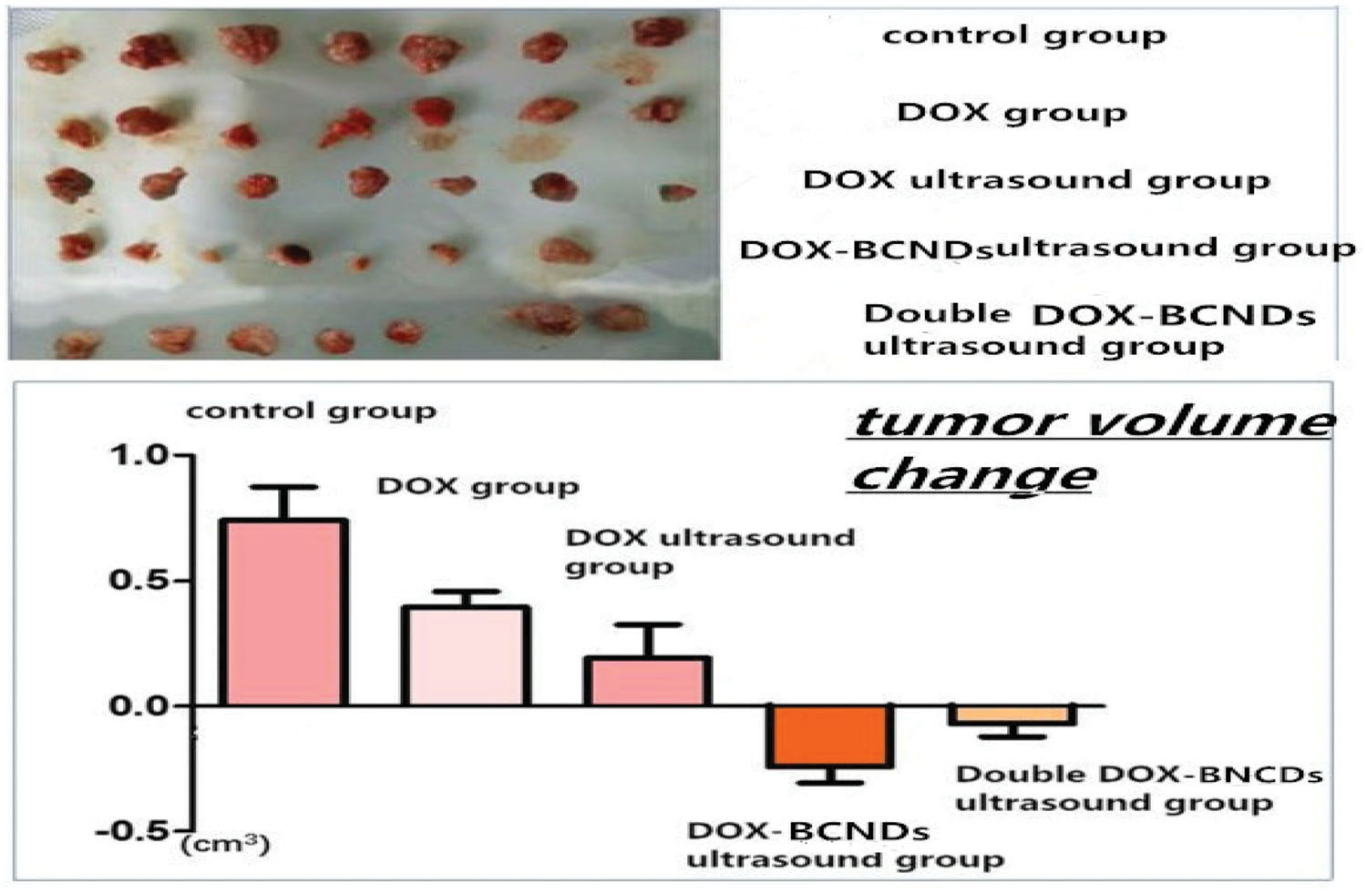
Gross anatomy of the tumors and tumor volume changes in the mice of each group.

The tumor volume increased more slowly in the DOX group and DOX ultrasound group than in the control group. The tumor volume decreased after treatment in the DOX-BCND ultrasound group and double DOX-BCND ultrasound group. The tumors in the DOX-BCNDs ultrasound group shrank more significantly than those in the double DOX-BCNDs ultrasound group (Table 3, Figure 6). These results indicate that DOX-BCNDs can promote the effects of DOX in the treatment of cancer. However, blindly increasing the dosage of DOX-BCNDs does not necessarily have a better therapeutic effect.

As shown in Figure 6, after treatment, the size of the tumors in the control group was larger than that in the other groups, while the sizes of the tumors in the DOX group and DOX-ultrasound group were larger than that in the DOX-BCNDs ultrasound group. At the same time, the size of the tumors in the double DOX-BCND ultrasound group was larger than that in the DOX-BCND ultrasound group. This result is consistent with the size of tumors measured by ultrasound.

The antitumor rates in the DOX group and DOX ultrasound group were 8.35% and 15.52%, respectively. The inhibition rate of the DOX-BCND ultrasound group was 39.50%, which was more than twice as large as that of the DOX group. The inhibitory rate of the double DOX-BCND ultrasound group was 30.73%. The results showed that the inhibition rate of the double DOX-BCND ultrasound group was lower than that of the DOX-BCND ultrasound group.

### 3.7. Blood biochemical results in mice

**Figure 7.**
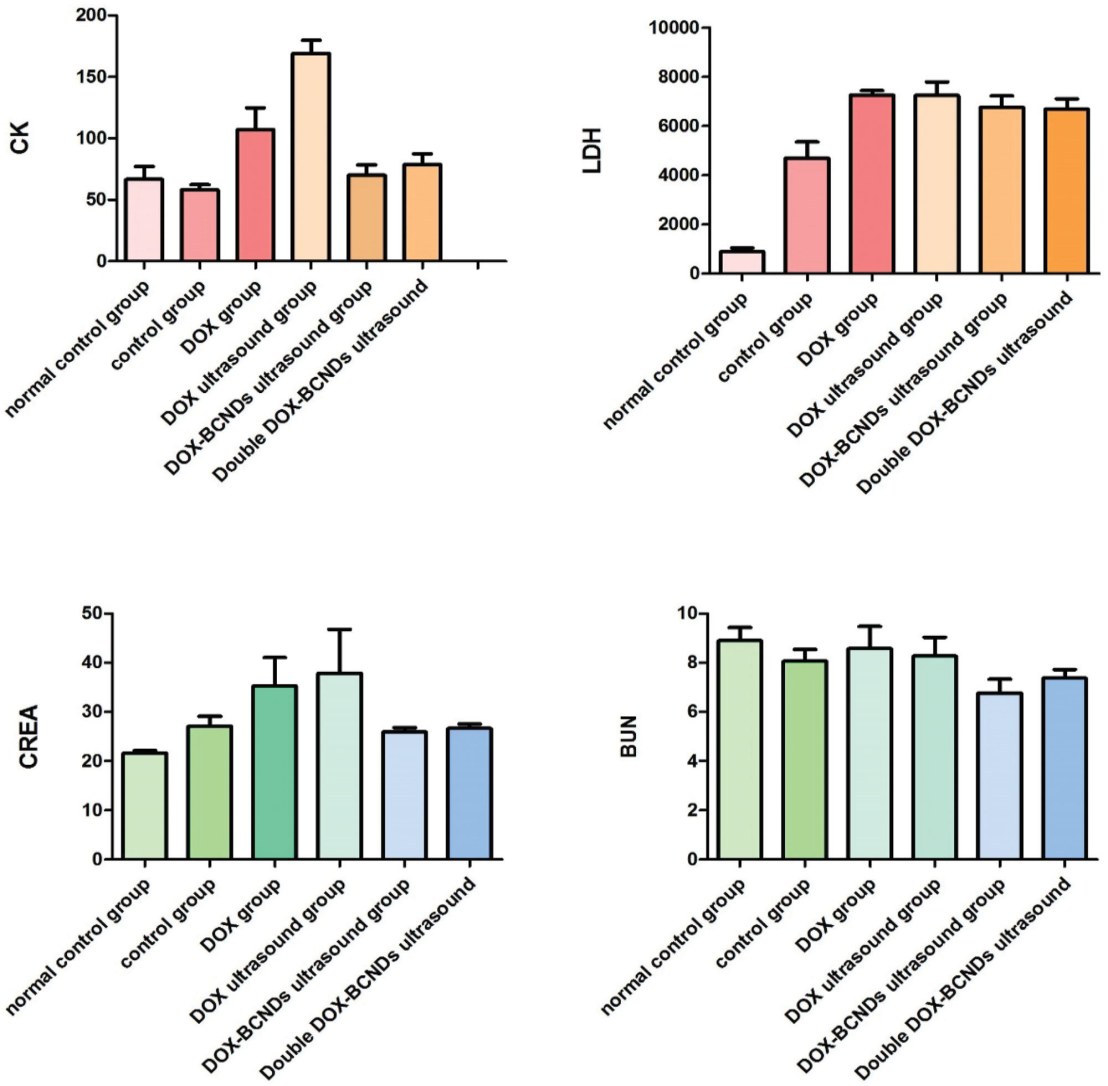
Comparison of CK, LDH, CREA, and BUN levels among the groups.

The results of the blood biochemical tests showed that there were differences among the groups in these values, including CK, LDH, CREA, and BUN (Table 4 and Figure 7). Comparative analysis within the six groups showed that CK in the DOX group was significantly higher than that in the control group and the DOX-BCNDs ultrasound group. CK in the DOX-ultrasound group was higher than that in the DOX group. These results suggested that the side effects of DOX included heart damage. However, in the DOX-BCND ultrasound group, DOX-induced cardiac injury could be reduced to a certain extent. The level of LDH in the DOX-BCNDs ultrasound group was slightly lower than that in the DOX group, but there was no significant difference.

**Table 4.** Comparison of the levels of CK, LDH, CREA, and BUN among the groups (mean ± SD).

Groups	CK (U/L)	LDH (U/L)	CREA (µmol/L)	BUN (mmol/L)
Control group	58.29 ± 10.53	4691.57 ± 1747.25	27.0 ± 5.30	8.06 ± 1.27
DOX group	107.14 ± 46.73**◆**	7255.14 ± 504.18**◆**	35.3 ± 15.26	8.57 ± 2.41
DOX ultrasound group	169.14 ± 28.61◆*	7239 ± 1442.97**◆**	37.8 ± 23.68	8.27 ± 2.03
DOX-BCNDs ultrasound group	70 ± 22.36*▼	6756.14 ± 1237.63**◆**	25.89 ± 2.50	6.76 ± 1.51
Double DOX-BCNDs ultrasound group	78.71 ± 22.86**▼**	6683.43 ± 1107.35**◆**	26.64 ± 2.40	7.37 ± 0.96

Notes: **◆**Compared with the control group, *p* < .05; *Compared with DOX group, *p* < .05; **▼**Compared with the DOX ultrasound group, *p* < .05.

The levels of CREA and BUN in the DOX group and the DOX ultrasound group were higher than those in the control group and the DOX-BCNDs ultrasound group, but the difference was not statistically significant. However, some data were not significantly different (*p* >  .05). We consider that this may be related to individual differences in the mice and the number of samples studied.

There was no significant difference in the results of the other blood biochemical indicators, including ALT, AST, TP, and ALB, among the groups (Table 5). This may also be related to liver damage caused by the H22 tumor itself.

**Table 5.** Comparison of the levels of ALT, AST, TP, and ALB among the groups (mean ± SD).

Group	ALT (U/L)	AST (U/L)	TP (g/L)	ALB (g/L)
Control group	64.57 ± 12.07	462.43 ± 123.93	53.23 ± 3.62	24.65 ± 3.95
DOX group	79.86 ± 25.06	546.29 ± 127.58	55.31 ± 1.02	27.21 ± 1.41
DOX ultrasound group	87.71 ± 30.58**◆**	612.43 ± 401.75	51.73 ± 3.91	23.23 ± 2.02*
DOX-BCNDs ultrasound group	84.57 ± 23.94	604.00 ± 108.56	53.47 ± 3.54	24.79 ± 2.16
Double DOX-BCNDs ultrasound group	66.17 ± 11.75	504.34 ± 155.79	54.40 ± 6.64	22.70 ± 2.22*

Notes: **◆**Compared with the control group, *p* < .05; *Compared with the DOX group, *p* < .05.

### 3.8. Routine blood test

DOX has the negative effect of reducing white blood cells (To et al., 2004). However, as shown in Table 6, this phenomenon was not evident in the DOX, DOX-ultrasound, and DOX-BCND ultrasound groups. However, the leucocyte levels in the double DOX-BCND ultrasound group with the higher DOX dose was significantly decreased compared with the other treatment groups.

**Table 6.** Comparison of routine blood indices among the groups (mean ± SD).

Group	White blood cell (×10^9^/L)	Lymphocyte (%)	Monocyte (%)	Granulocyte (%)	Red blood cell (×10^12^/L)	Erythrocyte (g/L)
Control group	6.53 ± 1.77	50.23 ± 15.10	13.30 ± 4.22	36.47 ± 12.62	8.38 ± 0.38	128.57 ± 7.57
DOX group	8.80 ± 2.30**◆**	64.87 ± 18.59	11.84 ± 5.94	23.29 ± 14.19**◆**	8.29 ± 0.49	131.71 ± 7.54
DOX ultrasound group	6.09 ± 1.70*	48.76 ± 15.43*	18.79 ± 1.37◆*	32.46 ± 15.04	7.70 ± 0.86	127.43 ± 18.12
DOX-BCNDs ultrasound group	7.14 ± 1.70	68.21 ± 9.76**◆▼**	10.73 ± 4.69▼	21.20 ± 6.94**◆**	7.86 ± 0.53	122.57 ± 7.96
Double DOX-BCNDs ultrasound group	5.39 ± 1.78*	55.43 ± 12.79	11.76 ± 2.13▼	32.81 ± 11.47	7.78 ± 1.14	123.29 ± 21.16

Notes: **◆**Compared with the control group, *p* <  .05; *Compared with the DOX group, *p* <  .05; **▼**Compared with the DOX ultrasound group, *p* <  .05.

### 3.9. He staining of paraffin sections and apoptotic experiments

**Figure 8.**
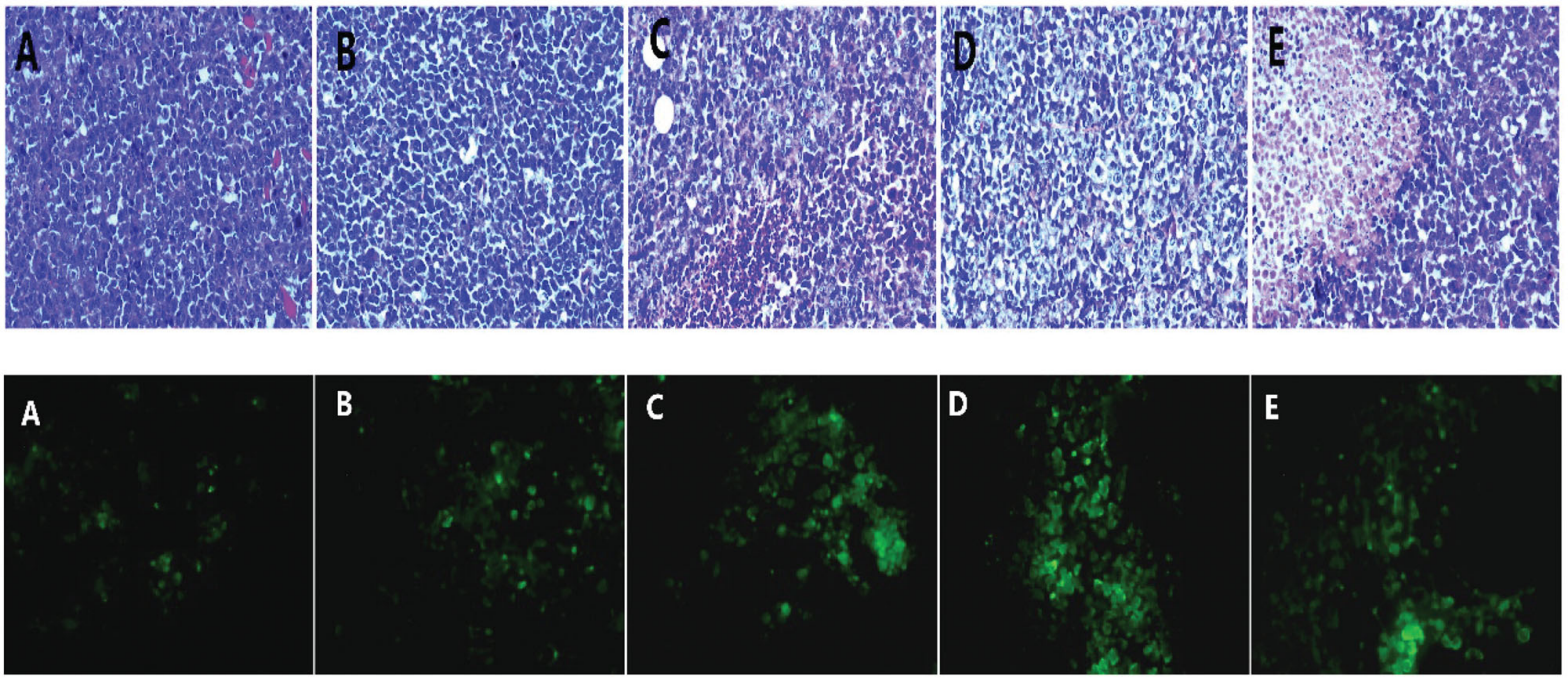
Paraffin section of tumor tissue with HE staining and Alexa Fluor 488 expression in tumor tissue observed under a fluorescence microscope.

HE staining showed that the nuclei of the tumor cells in the control group were amorphous and atypical with different cell sizes, eosinophilic cytoplasm, increased nucleosomes, and irregular cell arrangement (Figure 8). In the DOX group, vacuoles, degeneration, and necrosis were observed in some tumor cells. In the DOX-ultrasound group, the tumor cells were vacuolated and degenerated, and the number of necrotic and broken cells were slightly increased. Many tumor cells degenerated and necrotized in the DOX-BCND ultrasound group and the double DOX-BCND ultrasound group with obvious cell fragmentation.

The main mechanism of action of DOX treatment on tumors is the induction of apoptosis of the tumor cells. Figure 8 shows that the number of apoptotic tumor cells in the DOX group and the DOX ultrasound group was larger than that in the control group. The number of apoptotic cells in the DOX-BCNDs group and the double DOX-BCNDs group increased even more significantly. This phenomenon indicated that DOX-BCNDs promoted the apoptotic effect of DOX in tumor cells.

### 3.10. Immunohistochemical analysis

**Figure 9.**
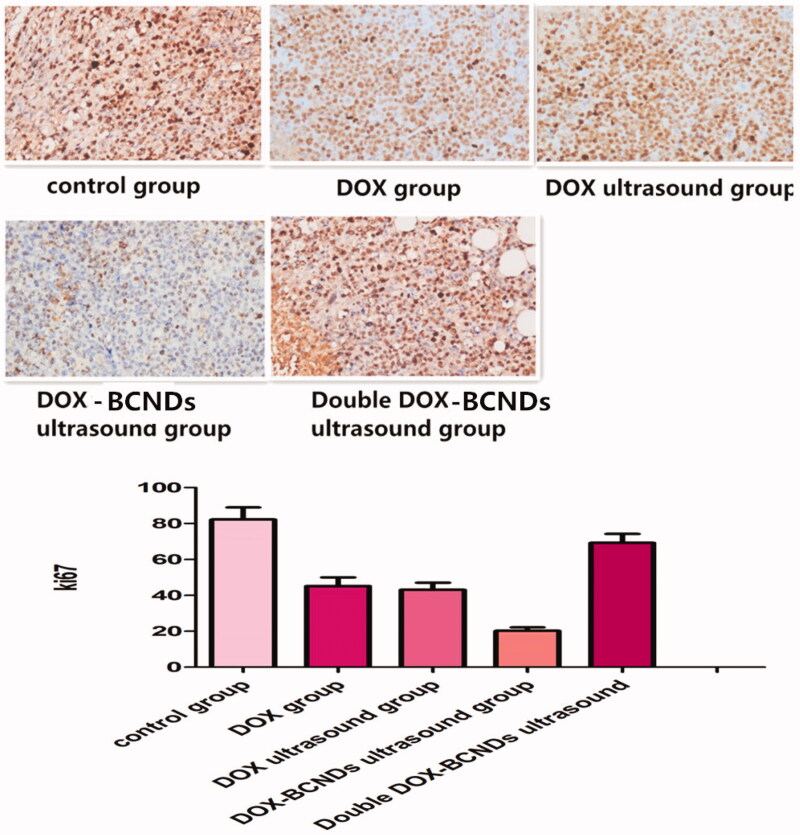
Immunohistochemical (IHC) detection of Ki67 in paraffin sections of tumors.

Cell proliferation was assessed in tumors using the monoclonal antibody Ki67. An Image-Pro Plus professional image analysis system (Media Cybernetics Inc., Bethesda, Maryland, USA) was used to measure the area and the average optical density. Ki67 immunohistochemistry staining showed that compared with the control group, the number of Ki67-positive cells decreased significantly in the DOX group and the DOX ultrasound group (*p* < .05), while the number of Ki67-positive cells decreased more significantly in the DOX-BCND ultrasound group (*p* < .05) (Figure 9), indicating that the application of DOX-BCNDs could significantly inhibit the proliferation of tumor cells. The expression of Ki67 in the double DOX-BCND group was higher than that in the DOX-BCND group, which may be related to the low antitumor immunity of the mice in this group. We will discuss this phenomenon later in this article.

## 4. Discussion

For safe and effective therapy, drugs are supposed to be actively targeted at the desired disease locations (Zhang et al., 2018). Nanodrug delivery systems have always been a research hotspot for targeted therapy. All kinds of nanoparticles made by proteins, macromolecules or other materials have been shown to have positive therapeutic value (Xin et al., 2017; Zhang et al., 2019a,b). However, the possible biological toxicity and sensitization of medical macromolecule polymers used in drug delivery systems need to be solved before they can be clinically applied (Nouman et al., 2017). Chitosan is a natural polysaccharide that commonly occurs in nature. A large number of studies on chitosan have shown that it has the characteristics of high biocompatibility and biodegradability, as well as antimicrobial and antitumor properties (Maeda & Kimura, 2004; Pang et al., 2017). Because of its antibacterial, antitumor and other characteristics, chitosan has attracted extensive attention from the medical community (Li et al., 2018). Palmitic acid and Epikuron 200 are also high-safety materials. To determine whether BCNDs are highly biocompatible, we performed an *in vivo* safety test of these chitosan nanodroplets using large doses of BCNDs. These results are encouraging because BCNDs have high biosafety.

In this study, the average diameter of the BCNDs was 519.6 nm, which could reach the tumor essence through the endothelial space of the neovascularization of tumor tissue by passive targeting and accumulating locally. We found that the application of perfluorohexane improved not only the stability of contrast agent, but also the yield of contrast agent. Chitosan, the main material of BCND shells, is rich in carboxyl and amino groups and carries positive charges. This lays the foundation for BCNDs to carry various small molecular substances or genes with negative charges in the future.

**Figure 10.**
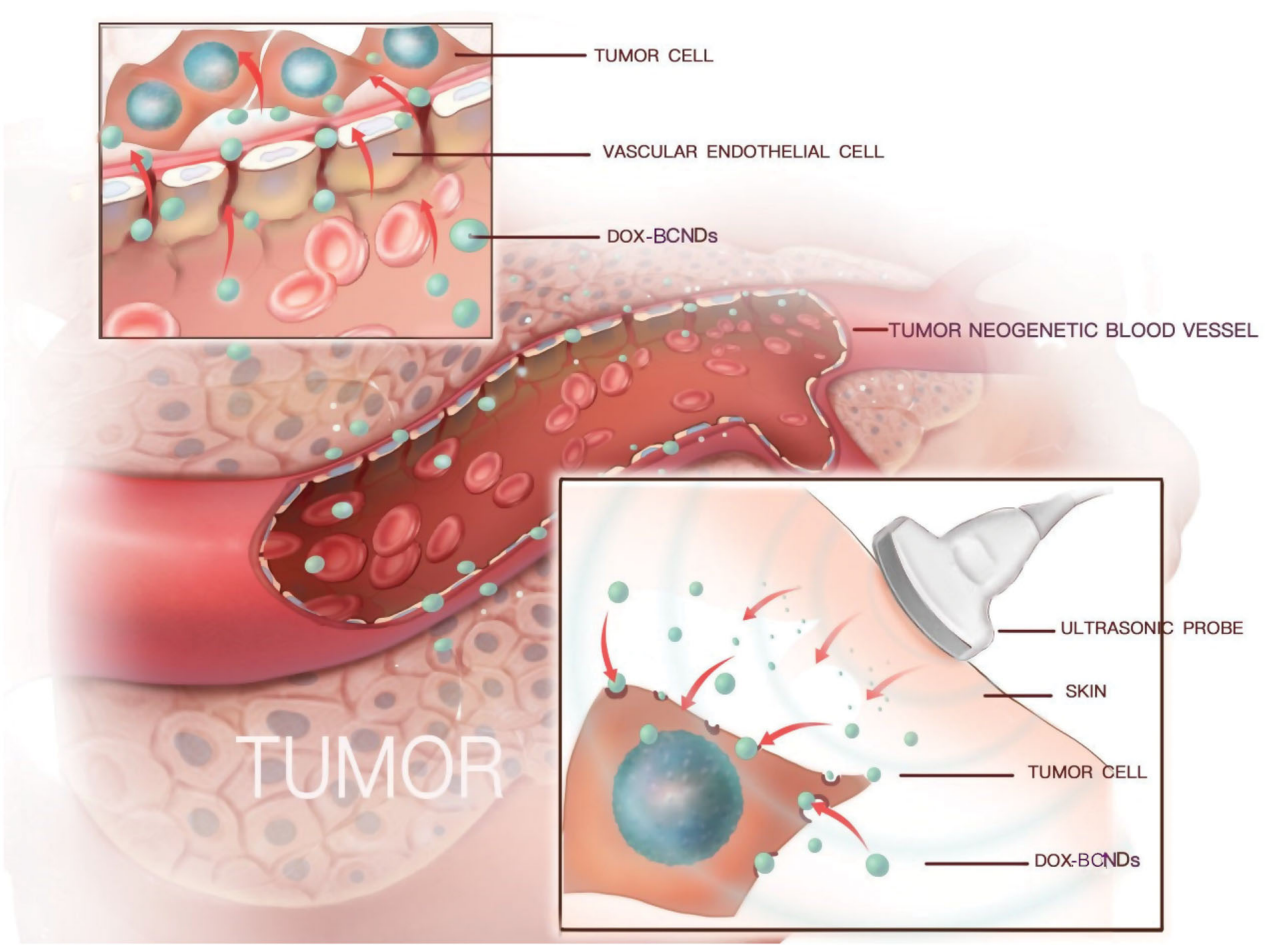
Mechanism of DOX-BCNDs entering cancer cells.

The mechanism of nanocarrier-targeted therapy is to enter the lesion area from the circulation through passive or active targeting. Passive targeting is mainly achieved through enhanced permeability and retention (EPR) effects (Zhang et al., 2019c). Then, nanocarriers can enter the target cells by endocytosis or other patterns, such as ultrasound-targeted microbubble destruction (UTMD) (Figure 10). Some studies have shown that treatment with doxorubicin-loaded nanometers with sonication results in better control of tumor growth than conventional treatment with doxorubicin injection or sonication (Hasanzadeh et al., 2013; Pan et al., 2016). In our research, BCNDs achieved the same effect.

DOX is one of the most effective anthracycline antitumor drugs that can interfere with DNA through insertion and then induce cancer cell apoptosis. However, its side effects on the heart, kidneys and other tissues are evident. Targeted therapy will help to increase its efficacy and reduce its side effects (Ahmed, 2016; Yoshizawa et al., 2016). In this study, DOX was loaded into BCNDs as an example drug and applied to cancer-targeting therapy. Our research showed that BCNDs had good imaging ability both *in vivo* and *in vitro* at 37 °C. This temperature is much lower than the boiling temperature of PFH, which further confirms that ultrasound plays a major role in the vaporization of the droplets (Baghbani et al., 2016).

The *in vivo* fluorescence imaging experiments in small animals and pharmacokinetics showed that DOX-BCNDs could significantly prolong the local aggregation time of DOX in tumors. This also slows down the diffusion of DOX to the surrounding tissues. This method effectively increases the efficacy of DOX and reduces its side effects. BCNDs play a protective role on the heart, kidney, and other tissues. This conclusion is similar to that of other nanotube drug delivery systems (Dong et al., 2017; Zhang et al., 2019c). In addition to its therapeutic effect on tumors, drug-loaded nanodrops also show a protective trend for other organs. The nanodrops tend to reduce the side effects of DOX. The reason may be attributed to the combination of nanodroplets and ultrasound to form ultrasound-targeted microbubble destruction (UTMD), which can instantly produce sound holes on the surface of tumor cell membranes (Lin et al., 2018). In terms of tumor inhibition rate, tumor pathology and immunohistology, DOX-BCNDs have shown a remarkable promoting effect on treatment with DOX. This conclusion is similar to the effects of other nanoparticles in previous literature (Gong et al., 2016).

In our study, we tried to increase the injection dose of DOX-BCNDs to observe the therapeutic effect of DOX. It was interesting to find that increasing the injection dose under certain conditions did not significantly increase the efficacy. Although the tumor volume of mice in the double DOX-BCND ultrasound group decreased, the expression of Ki67 in H22 tumor cells in this group increased. We found that the tumors in the double DOX-BCND ultrasound group were softer than those in the other groups, and HE staining showed that there were more necrotic tissues in these tumors. We inferred that the reason for the decrease of tumor volume in the double DOX-BCND ultrasound group may be the increase of liquefaction and necrosis in tumor tissue. Whether the necrosis of tumor tissue is related to the dense cavitation effect of ultrasound irradiation while injecting large numbers of nanodroplets into the tumor site remains to be confirmed.

Moreover, the number of leukocytes in the double DOX-BCND ultrasound group was significantly lower than that in the other groups. Therefore, we believe that the decrease of tumor inhibition in the double DOX-BCND ultrasound group is related to leukocytopenia in peripheral blood and decreased immunity (Joseph et al., 2014). In addition, some mice in the double DOX-BCND ultrasound group suffered from loss of appetite, mental retardation, and weight loss, which may be related to the damage of intestinal endothelial cells induced by DOX (Christopher & Dekaney, 2009). The side effects of an excessive dose of DOX may promote deterioration of the mouse body condition and reduce their own anticancer ability. The results showed that to achieve a better therapeutic effect for different types of drug-loaded nanocarriers, doses given at less than half the lethal dose were not sufficient. The dose of drug-loaded nanocarriers used in the clinic still needs to be determined through experimental studies.

## 5. Conclusion

Our study is the first to evaluate the safety, imaging and therapeutic effects of BCNDs *in vivo*. The results showed that as a novel and highly biocompatible ultrasound contrast agent, BCNDs had good imaging and promoting effects of DOX *in vivo*. Moreover, BCNDs showed a trend to protect other tissues of the body. The highly biocompatible BCNDs have been proven to be an effective strategy in cancer treatment.
